# Sero-epidemiological study in prediction of the risk groups for measles outbreaks in Vojvodina, Serbia

**DOI:** 10.1371/journal.pone.0216219

**Published:** 2019-05-09

**Authors:** Mioljub Ristić, Vesna Milošević, Snežana Medić, Jelena Djekić Malbaša, Smiljana Rajčević, Jasmina Boban, Vladimir Petrović

**Affiliations:** 1 University of Novi Sad, Faculty of Medicine, Department of Epidemiology, Novi Sad, Serbia; 2 Institute of Public Health of Vojvodina, Centre for Disease Control and Prevention, Novi Sad, Serbia; 3 University of Novi Sad, Faculty of Medicine, Department of Microbiology with Parasitology and Immunology, Novi Sad, Serbia; 4 University of Novi Sad, Faculty of Medicine, Department of Radiology, Novi Sad, Serbia; 5 Oncology Institute of Vojvodina, Centre for Diagnostic Imaging, Sremska Kamenica, Serbia; ESIC Medical College & PGIMSR, INDIA

## Abstract

**Background:**

Age-stratified serologic surveys provide insight into the gaps of measles-specific immunity as well as estimates of the age-specific seroprevalence. The aim of this study was to describe the measles sero-epidemiology in Vojvodina before the occurrence of outbreak in 2017/18 and to discuss preventive measures for potential future epidemics.

**Methods:**

A seroprevalence study was conducted from April 2015 to June 2017 on serum bank of 3199 residual samples. Study was performed prior to the last measles outbreak in Vojvodina that occurred between 12th November 2017 and 30th June 2018. Measles-specific IgG antibodies were determined using an indirect chemiluminescent immunoassay (CLIA).

**Results:**

Median age of enrolled participants was 20 years (IQR 11–37). Overall, 86.9% serum samples were seropositive. The highest proportion of measles seronegativity was observed in children aged 12–23 months of age and in adults aged 20–39 years (56.1% and 18.5%, respectively). Prevalence of measles seronegativity above WHO target levels susceptibility was observed in the following age groups: 2, 7, 13, 15, and among all adults aged between 20 and 49 years. Out of total measles outbreak cases (177), there were 91 (51.4%) participants aged 20–39 years. A significant positive correlation was observed between measles seronegativity and the number of reported measles cases aged ≥ 12 months (r = 0.4675, p = 0.0213).

**Conclusions:**

In order to prevent new outbreaks and achieve the elimination of measles in Vojvodina, the vaccination coverage of both measles-mumps-rubella (MMR1 and MMR2) vaccines needs to be improved and sustained. Educational campaigns for the improvement of acceptance and timely vaccination with vaccine against measles among doctors and the general population are crucial. Our results indicate possible gap in measles protection in adults born during implementation of one dose of measles vaccine and prioritize supplementary immunization activities targeting adults in Vojvodina, Serbia.

## Introduction

Measles is a highly contagious viral disease with high potential to circulate even in the countries with low population susceptibility rate [1, 2]. An estimated 7 million people were affected by measles in 2016 globally. In addition, between 2000 and 2016, the annual reported measles incidence decreased by 87%, while global measles mortality decreased by 84%, with an estimated 20.4 million measles-associated deaths prevented [1]. The World Health Organization (WHO) has targeted measles for elimination by 2020, which seems to be a hardly achievable goal [3–5]. Worldwide progress towards the reduction in the number of measles cases and deaths stagnated between 2008 and 2010 [6, 7], largely due to numerous prolonged measles outbreaks in Africa, India, and in the western European countries [8]. Since 2005, Serbia has been included into the strategic plan for the elimination of measles and congenital rubella syndrome in the European Region, supported by the WHO [9, 10].

Before the introduction of mandatory measles immunization in Serbia in 1971, large measles outbreaks in the Autonomous Province of Vojvodina (Vojvodina) were initially recorded every 1–3 years, with predominantly preschool children being infected [11]. During the vaccine era, measles incidence in Vojvodina dropped dramatically. In the period between 2001 and 2006 there were no measles cases registered [11]. Small measles outbreak with 200 measles cases, mostly among unvaccinated hard to reach Roma children occurred in 2007 in Vojvodina [12]. Later, during the 2014/15, a total of 93 measles cases were notified among predominantly unvaccinated students of the University of Novi Sad from Bosnia and Herzegovina [11]. The last outbreak of measles in Vojvodina, started in 2017 and represents the part of the largest outbreak of measles in Serbia during the last 25 years [13]. Meanwhile, a total of 41,000 measles cases, including 37 deaths occurred in the first six months of 2018, in seven European countries [14].

Determining a country-specific immunization strategy requires knowledge of the unique population susceptibility profile. Age-stratified serosurveys provide insight into the gaps of measles-specific immunity as well as estimates of the age specific seroprevalence [15–17]. To date, there are no available data on measles seroprevalence in Serbia.

The aim of this study was to describe the measles sero-epidemiology in Vojvodina before the occurrence of the outbreak in 2017/18 and to discuss future preventive measures.

## Materials and methods

### Description of the notification system and immunization programme development

Mandatory notification of measles in Vojvodina—the northern Autonomous Province of Serbia with a population of 1,931,809 (representing 26.9% of the total Serbian population)—has been conducted since 1948. Mandatory measles immunization with a single monovalent dose was introduced in 1971 targeting all children aged 12–15 months. Combined measles-mumps (MM) vaccine became available in 1986. In 1993, the MM vaccine was replaced with a combined measles-mumps-rubella (MMR) vaccine. In 1994, a two-dose schedule using MMR at age 12–15 months and 12 years was implemented. Second dose was moved to preschool age (6–7 years) in 2006 [11].

### Study population and survey design

This cross-sectional serosurvey of measles-specific IgG antibodies was conducted in the Centre for Diseases Control and Prevention, and Centre for Virology of the Institute of Public Health of Vojvodina (IPHV), Novi Sad. The collection of the main serum bank was performed according to the guidelines and specifications of the European Sero-Epidemiology Network 2 (ESEN2) project [18]. Details of the survey design and sampling method have been described previously [19, 20]. In brief, a total of 3199 residual diagnostic sera from participants were collected during inter-epidemic period in Vojvodina, from April 2015 to June 2017 (point of seroprevalence survey). The serum bank comprised anonymised residual diagnostic samples from manifestly healthy subjects without any signs or symptoms of acute illness on routine blood count aged 12 months and older. Written informed consent was obtained from each participant before enrolment. For participants aged < 15 years, they were explained the study objectives and procedures according to their level of understanding, and consent from their parents or legal guardians was obtained. Personal and confidential information were removed, except for demographic information, including date of sampling, settlement area, age and sex of participants. In order to estimate general population immunity, collected serum samples were representative regarding geographical area, age group and sex in Vojvodina. According to their residential addresses, participants from 45 municipalities of Vojvodina were divided into seven Districts: North Bačka, West Bačka, South Bačka, North Banat, Central Banat, South Banat, and Srem. We obtained only individual immunization data from current measles outbrek cases, and there were no individualized immunization data for subjects included in the serosurvey study (only coverage immunization data of the vaccine against measles available from registers of immunization from seven Districts of Vojvodina were obtained).

### Specimen collection and serology

Blood samples were collected aseptically using venipuncture technique by qualified personnel. Upon collection, serum samples were stored in a refrigerator (2–8°C), and transported to the laboratory of the Centre for Virology of the IPHV in accordance with the principles of cold chain. For each sample, serum was separated using a centrifuge and stored at –20°C until assayed. Serums were tested according to the manufacturer’s instructions. We predicted that the hyperlipemic, hemolysed or contaminated samples as well as the samples of immunocompromised patients and recipients of blood or blood products during the past 6 months before sampling were excluded from further sera testing.

Measles-specific IgG antibodies were determined using an indirect semi-quantitative chemiluminescent immunoassay (CLIA) (Measles VIRCLIA IgG Monotest, **Vircell S.L.U.,** Parque Tecnológico de la Salud., Calle Avicena, 8, 18016 Granada, Spain), in accordance with the manufacturer’s instructions. In our study, measles serostatus was determined by following standard protocols with sample Antibody index (AI) value cutoffs determined by the manufacturer: greater than 1.1 AI/ml was considered positive (protected against measles), less than 0.9 AI/ml was deemed negative (susceptible to measles), and between 0.9 and 1.1 AI/ml was grouped equivocal (unknown measles immunity status). Results of all tested residual samples were classified based on the regional affiliation (Districts of Vojvodina), settlement area (urban or suburban/rural), sex and age of participants.

### Case definitions and data source

The ongoing measles outbreak in Vojvodina during 2017/18 was analysed in relation to the obtained measles seroprevalence data. Similar to the methodology that was used in previously conducted research [21], final classifications of measles cases were done according to the WHO criteria [9]. In accordance with previously described procedures [22], laboratory confirmation of measles was performed in the WHO Measles/Rubella National Reference Laboratory at the Institute of Virology, Vaccines and Sera “TORLAK” Belgrade. Measles surveillance data i.e. notified number of cases collected between 1948 and 2017, including measles vaccine coverage rate in Vojvodina, as well as data related to the ongoing measles outbreak (November 2017-June 2018) were obtained from the surveillance database of the Centre for Disease Control and Prevention of IPHV.

The annual incidence rates of measles were measured per 100,000 inhabitants. The numerator was the number of the reported measles cases in the total population of Vojvodina. The denominator was the whole population monitored during each consecutive year (1948–2017). Vaccination coverage in our country is monitored by the administrative method [23]. To estimate the annual coverage rates of immunization against measles in Vojvodina, the total number of immunized children (numerator) within one calendar year in a certain area (municipality, District, Province) was divided by the total number of children who should have been immunized according to their age by Serbian immunization schedule (denominator). The data on immunization coverage from immunization records of children were obtained from Government Health Care Centres across Vojvodina annually, as a part of routine surveillance of mandatory immunization in Vojvodina [11].

Seroprevalence of study subjects were analysed in relation to the birth cohorts and measles immunization policy at the time: pre-immunization period for cohorts born between 1931 and 1970; single measles monovaccine period (cohorts born from 1971 to 1985); single combined measles-mumps vaccine period (cohorts born between 1986 and 1993), and the two doses combined measles-mumps-rubella vaccine period (cohorts born since 1994).

### Statistical analysis

Statistical analysis was performed using SPSS software tool (version 22.0) MedCalc for Windows, version 12.3.0 (MedCalc Software, Mariakerke, Belgium).

We calculated proportions, geometric mean concentration (GMC) and 95% confidence intervals (CIs) of measles seroprevalence in the study population at the point of seroprevalence survey. Analysis of variance (ANOVA) was used to compare the GMC by age and sex of participants. The association between the dependent variable, measles seronegativity (excluding equivocal results) and the independent variables, including District, settlement area, sex, and age, were analyzed using logistic regression modelling (univariate and multivariate analysis). The correlation was calculated using Spearman’s correlation coefficient. Statistical significance was set at p<0.05.

### Ethical consideration

The study protocol was reviewed and approved by Medical Ethics Committee of Institute of Public Health of Vojvodina, on 14 of May 2015 under the number 01-79/7a, as a part of wider serosurvey on vaccine preventable diseases in Vojvodina.

## Results

**Fig 1:**
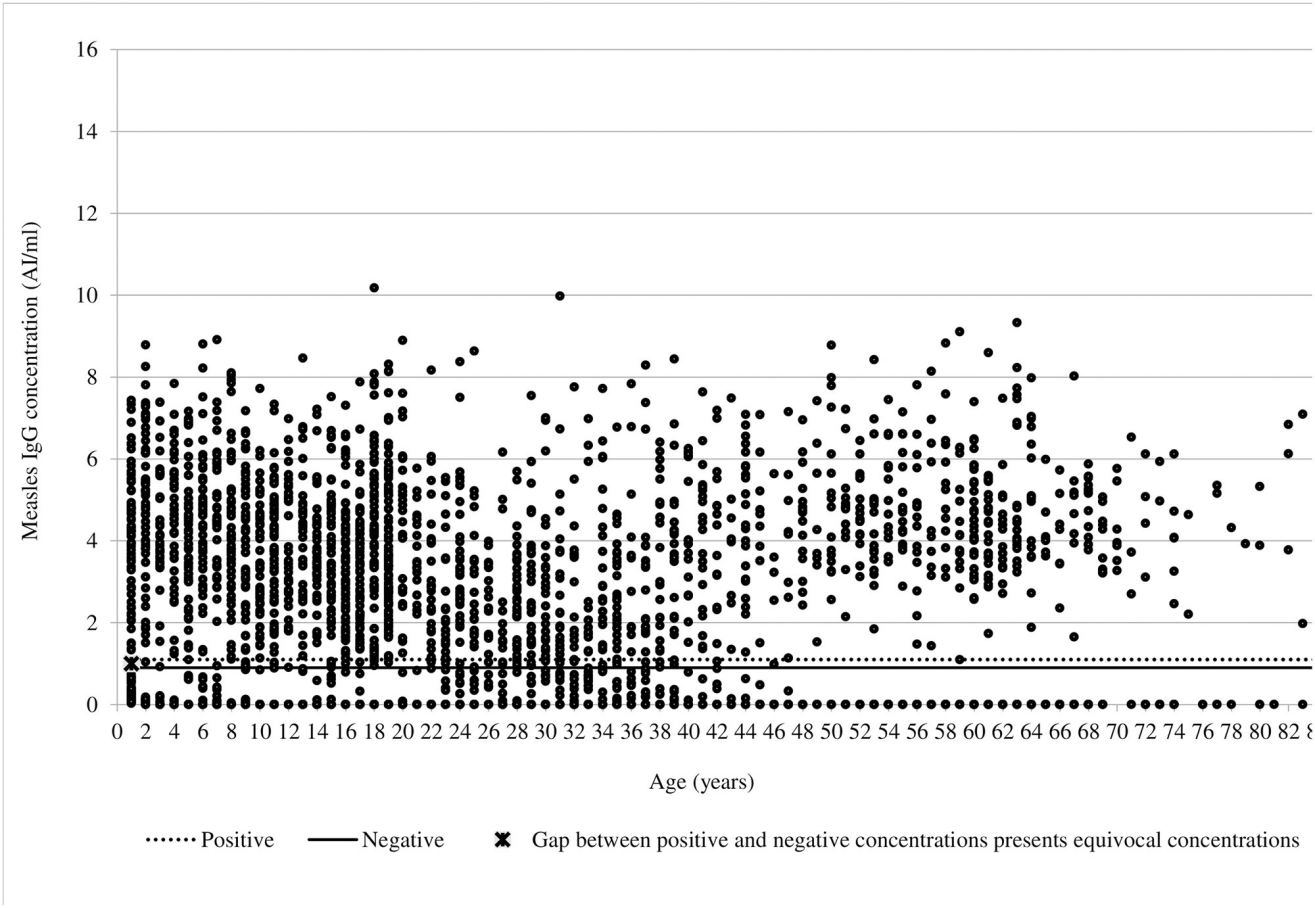
Scatter plot of measles IgG concentration (AI/ml) by age in Vojvodina, Serbia, 2015–2017.

The original sample of the varicella seroprofiles of Vojvodina, the first part of the seroprevalence project, included 3570 subjects. A total of 3199 serum samples were available for this study. All subjects were born between 1931 and 2016. Overall, median age was 20 years (IQR 11–37), and 50.8% of all enrolled participants were females. The antibody index concentration of the study sample ranged from 0.002 to 10.176 AI/ml, and median value was 4 AI/ml (IQR 2–5). Most of the subjects (2780) had anti-measles IgG level above 1.1 AI/ml, which was considered positive (Fig 1).

### Seroprevalence of measles by cohorts, immunization coverage against measles and incidence rate of measles in Vojvodina, Serbia

**Fig 2:**
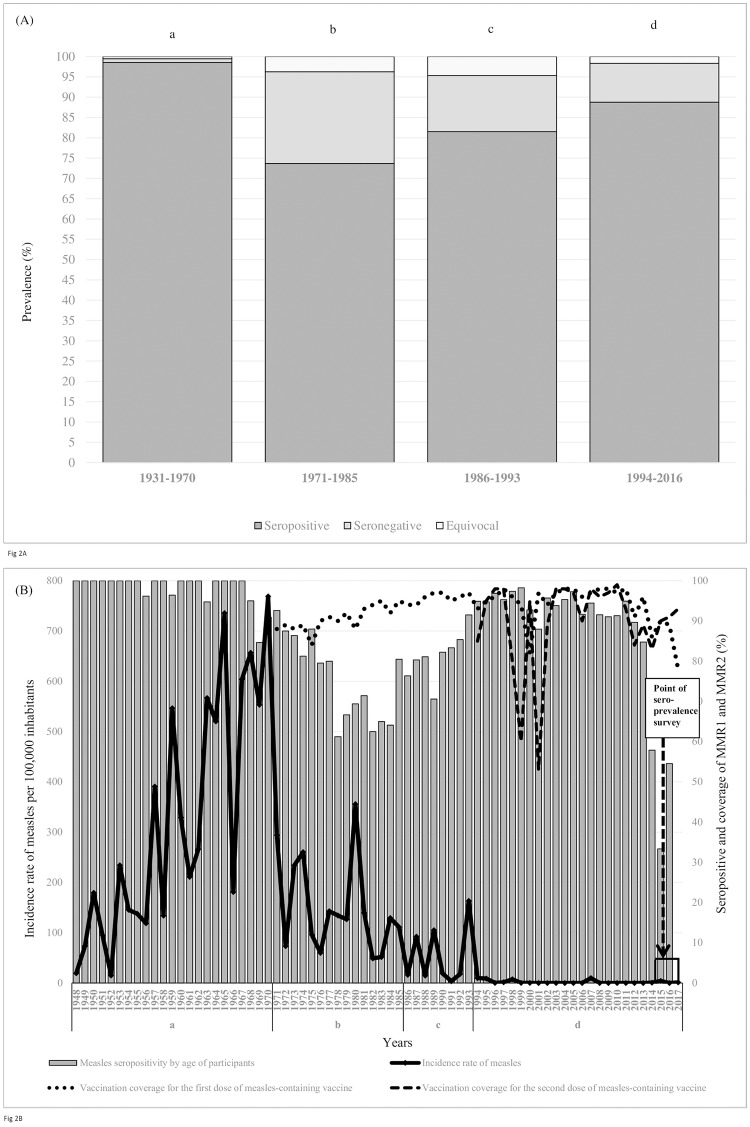
Seroprevalence of measles antibody in four birth cohorts (A), and measles incidence, measles seropositivity and MMR vaccine coverage rates (B) in Vojvodina, Serbia. a-vaccine-free period; b-single measles monovaccine period; c-single combined measles-mumps vaccine period; d-two doses combined measles-mumps-rubella vaccine period.(B) x-axis represents actual years for measles notification per 100,000 inhabitants, measles seropositivity by age considering the point prevalence of the study (time of sampling 2015–2017), and annual immunization coverage of the first and the second dose of measles vaccine for those born one year earlier than the year on x-axis.

Observed by seroprevalence data, during the pre-immunization period, average seropositivity to measles was 98.6%, then decreased to 73.7% during the single measles monovaccine period, and further increased to 81.5% and 88.8% during the single combined measles-mumps vaccine period and throughout the two doses combined measles-mumps-rubella vaccine period, respectively (Fig 2A). Aforementioned data can indicate that results of measles seropositivity at the point of prevalence in this study (2015–2017) reflect the herd immunity. This immunity can be considered as a result of previous naturally acquired infection (during a vaccine-free period) or as a result of naturally acquired or vaccine induced immunity (during single or two doses vaccine period). According to the available data, it was not possible to distinguish if naturally or vaccine induced immunity was measured at the point of this seroprevalence survey.

Declining trend of measles incidence in Vojvodina in 1948–2017 was observed with wide variation in annual notification rates from 768.8/100,000 in 1970 to 0 during 12 different period/years (2001–2006, 2008–2011, 2012, and 2016).

During the first ten years of one dose of measles vaccine period, the coverage rate of measles monovalent vaccine ranged between 84% and 90%. Probably due to accumulation of susceptible population, the largest measles outbreak was recorded in 1980 (annual notification rate of 355.6/100,000). Although the measles seropositivity during two doses of MMR vaccine period ranged from 33.3% (in 2015) to 98.2% (in 1999), the annual incidence rates of measles did not exceed 10.0/100,000. Between 1994 and 2017, the average coverage rates of both MMR1 and MMR2 vaccine were 93.8% and 89.5%, respectively. At the point of this seroprevalence survey, measles seropositivity was the lowest in the 2014–2016 period-participants aged 1–3 years (33.3%-57.9%) when the average coverage rate with both MMR1 and MMR2 vaccines was only 88.0%. In 2017, the lowest MMR1 coverage rate (78%) was observed in Vojvodina since its introduction in 1971. On the other hand, considering the point prevalence of this study (2015–2017), measles seropositivity ranged between 80% and 85% during the 1976–1992 period. This fact can possibly suggest that the subjects at the moment of sampling were aged 23–39 years (subject sampled in 2015) i.e. aged 25–41 years (participants sampled in 2017). Altough the average coverage rate of measles monovalent vaccine in this period was close to 93% (which influenced further decrease of the annual incidence rates of measles), the low measles seropositivity in these age groups at the point of this seroprevalence survey may reflect the waning of immunity against measles over time after administration of one dose vaccine as well as the possibility that some of them were not vaccinated against measles until this serosurvey or were not previously in contact with measles virus (Fig 2B).

### Coverage of vaccine against measles by Districts of Vojvodina, 1999–2017

According to available immunization data during the 19-year period (1999–2017), the vaccination coverage rates of MMR1 and MMR2 vaccine were different in seven Districts of Vojvodina. In the South Bačka District, the coverage of MMR1 in 2017 was only 61.6%, while the coverage of MMR2 in 2013 was 67%. During the aforementioned period, the immunization coverage of <95% for MMR1 or MMR2 in all seven Districts of Vojvodina was observed in at least four different years (S1 Table).

### Prevalence of IgG antibodies against measles by sociodemografic factors

A detailed overview of residual diagnostic seroprevalence of anti-measles IgG positive, equivocal, and negative results, as well as the geometric mean concentration (GMC) for all tested participants is shown in Table 1.

**Table 1:** Seroprevalence and geometric mean concentration of measles IgG antibodies by District, settlement area, sex, and age groups in Vojvodina, Serbia, 2015–2017.

Characteristics		No of samples	Proportion seropositive % (95% CI)	Proportion equivocal % (95% CI)	Proportion seronegative % (95% CI)	Geometric mean concentration AI/ml (95% CI)
District of Vojvodina	North Bačka	239	91.21 (86.88–94.48)	1.67 (0.46–4.22)	7.12 (4.20–11.15)	3.03 (2.79–3.27)
	West Bačka	223	87.44 (82.36–91.49)	2.69 (0.99–5.76)	9.87 (6.29-14-56)	2.66 (2.41–2.91)
	South Bačka	1347	85.60 (83.61–87.43)	1.48 (0.91–2.28)	12.92 (11.17–14.83)	2.77 (2.64–2.90)
	North Banat	269	86.25 (81.55–90.13)	2.60 (1.05–5.28)	11.15 (7.65–15.53)	2.66 (2.44–2.88)
	Central Banat	244	86.07 (81.08–90.16)	3.69 (1.70–6.89)	10.24 (6.74–14.75)	2.69 (2.46–2.92)
	South Banat	392	85.20 (81.29–88.57)	3.83 (2.16–6.24)	10.97 (8.05-14-49)	2.70 (2.49–2.91)
	Srem	485	90.31 (87.32–92.79)	1.86 (0.85–3.50)	7.83 (5.60–10.59)	2.94 (2.77–3.11)
Settlement area	Urban	1899	86.99 (85.39–88.47)	2.0 (1.42–2.74)	11.01 (9.64–12.50)	2.80 (2.70–2.90)
	Suburban or rural	1300	86.77 (84.81–88.57)	2.46 (1.69–3.46)	10.77 (9.14–12.58)	2.75 (2.64–2.86)
Sex	Male	1573	85.70 (83.87–87.39)	2.61 (1.88–3.52)	11.69 (10.14–13.38)	2.67 (2.56–2.78)
	Female	1626	88.07 (86.39–89.61)	1.78 (1.19–2.55)	10.15 (8.72–11.72)	2.89 (2.79–3.00)
Age in years	1 (12–23 months)	155	41.94 (34.07–50.12)	1.94 (0.93–7.06)	56.12 (47.93–64.07)	0.67 (0.33–1.01)
	2–4	187	87.70 (82.12–92.04)	1.07 (0.13–3.81)	11.23 (7.09–16.65)	2.97 (2.65–3.29)
	5–9	358	91.62 (88.25–94.27)	1.40 (0.46–3.23)	6.98 (4.57–10.13)	3.31 (3.01–3.61)
	10–19	886	95.37 (93.77–96.66)	1.92 (1.12–3.06)	2.71 (1.74–4.01)	3.34 (3.22–3.46)
	20–39	890	77.08 (74.18–79.80)	4.38 (3.13–5.94)	18.54 (16.04–21.25)	2.05 (1.92–2.18)
	≥40	723	95.71 (93.97–97.07)	0.55 (0.15–1.41)	3.74 (2.48–5.39)	3.98 (3.86–4.10)
Total		3199	86.90 (85.68–88.05)	2.19 (1.71–2.76)	10.91 (9.85–12.04)	2.78 (2.70–2.86)

Overall, 86.90% (95% CI 85.68–88.05), 2.19% (95% CI 1.71–2.76) and 10.91% (95% CI 9.85–12.04) serum samples were positive, equivocal and negative for measles-specific IgG antibodies, respectively. In total, the GMC of anti-measles IgG was 2.78 AI/ml (95% CI 2.70–2.86), and values of GMCs antibodies indicated a pattern similar to that found in measles seropositivity i.e. apart from the adults aged 20–39 years, GMC showed an upward trend with regard to the age group, showing the highest value of 3.98 AI/ml (95% CI 3.86–4.10) in the subjects aged ≥40 years.

The proportion of anti-measles IgG seronegative results was the highest in the South Bačka District (12.92%; 95% CI 11.17–14.83), while the proportion of measles seronegativity regarding settlement area (urban or suburban/rural) was similar (up to 11%). Proportions of measles seronegativity and equivocal were higher in males (11.69%; 95% CI 10.14–13.38 and 2.61%; 95% CI 1.88–3.52, respectively) compared to females (10.15%; 95% CI 8.72–11.72 and 1.78%; 95% CI 1.19–2.55, respectively), but the differences were not statistically significant (p>0.05).

Analysis according to age revealed that the highest proportion of measles seronegativity was present in children aged 12–23 months of age and in adults aged 20–39 years (56.12%; 95% CI 47.93–64.07 and 18.54%; 95% CI 16.04–21.25, respectively), while the lowest proportion of seronegativity to measles was among participants aged 10–19 years (2.71%; 95% CI 1.74–4.01).

Various sociodemographic characteristics were significantly associated with negative seroprevalence of anti-measles IgG in both the univariate and multivariate analysis (Table 2). Seronegativity to measles was significantly higher in the South Bačka compared with the North Bačka District (OR 1.94; 95% CI 1.15–3.25, p = 0.012; adjusted OR 1.79; 95% CI 1.06–3.01, p = 0.030) of Vojvodina. Using the 10–19 years age group as a comparator (the lowest measles seronegativity), after adjusting, the prevalence of measles seronegativity was significantly higher in the following age groups: children aged 12–23 months, 2–4 years, 5–9 years, and adults aged 20–39 years (OR 44.89, 95% CI 26.70–75.50; OR 4.33, 95% CI 2.34–8.02; OR 3.01, 95% CI 1.68–5.39, and OR 8.13, 95% CI 5.23–12.63, respectively). There were no statistically significant associations between measles seronegativity regarding sex or settlement area.

**Table 2:** Association between sociodemographic factors and the risk of a seronegative measles in Vojvodina, Serbia, 2015–2017.

Characteristics		crude OR (95% CI)	p value	adjusted OR ^a^^,^ ^b^ (95% CI)	p value ^b^
District of Vojvodina	North Bačka	Referent			
	West Bačka	1.43 (0.74–2.77)	0.290		
	South Bačka	1.94 (1.15–3.25)	0.012	1.79 (1.06–3.01)	0.030
	North Banat	1.64 (0.88–3.05)	0.120		
	Central Banat	1.49 (0.78–2.84)	0.224		
	South Banat	1.61 (0.90–2.89)	0.111		
	Srem	1.11 (0.61–2.01)	0.730		
Settlement area	Urban	1.02 (0.82–1.29)	0.833		
	Suburban or rural	Referent			
Sex	Male	1.17 (0.94–1.47)	0.160		
	Female	Referent			
Age in years	1 (12–23 months)	45.95 (27.46–76.90)	<0.001	44.89 (26.70–75.50)	<0.001
	2–4	4.54 (2.47–8.35)	<0.001	4.33 (2.34–8.02)	<0.001
	5–9	2.70 (1.52–4.79)	0.001	3.01 (1.68–5.39)	<0.001
	10–19	Referent			
	20–39	8.17 (5.27–12.68)	<0.001	8.13 (5.23–12.63)	<0.001
	≥40	1.39 (0.80–2.44)	0.245		

The group with the lowest seronegativity by characteristics was used as the reference.

^a^ Adjusted for the following variables: District, settlement area, sex, and age.

^b^ Identified risk factors for seronegativity with a p-value <0.05 in the univariable analysis were included in a multivariable logistic regression analyses.

### Prevalence of IgG antibodies against measles by age group and by sex

When stratified by age, the GMC of measles antibodies in girls was significantly higher compared to boys aged 3 and 11 years (p = 0.006 and 0.023, respectively), but significantly lower than among boys aged 4 years (p = 0.037) (S1 Fig).

### Baseline characteristics of measles outbreak in Vojvodina, Serbia

Between 12^th^ November 2017 and 30^th^ June 2018, a total of 177 measles cases with 87 males and 90 females were reported during measles outbreak, and there were no related deaths reported. The majority (82 of 177, 46.33%) of measles cases were registered in the South Bačka District, where the highest prevalence (12.9%) of measles seronegativity was recorded. Out of the total measles outbreak cases, there were 91 (51.4%) participants aged 20–39 years and 15 (8.5%) cases younger than 12 months of age. Apart from the North Bačka District, where the mean age of measles cases was 13 years (range 0–40 years), mean age of measles cases in all other Districts of Vojvodina ranged between 27 (South Bačka) and 39 (West Bačka) years. Mean age of the total measles outbreak cases was 29 years (range 0–56 years). The majority of the measles cases (97/177; 54.8%) in Vojvodina were previously vaccinated with one dose of measles-containing vaccine (MCV) (Table 3).

**Table 3:** Prevalence of measles seronegativity (2015–2017) and measles cases in outbreak 2017–2018 in Vojvodina, Serbia.

Variable		North Bačka	West Bačka	South Bačka	North Banat	Central Banat	South Banat	Srem	Vojvodina
Prevalence of measles seronegativity (%)		7.1	9.9	12.9	11.2	10.2	11.0	7.8	10.9
No (%) of measles cases in outbreak 2017/18		4 (2.26)	5 (2.83)	82 (46.33)	11 (6.21)	2 (1.13)	14 (7.91)	59 (33.33)	177
Mean age of measles cases in outbreak 2017/18 (years) (range)		13 (0–40)	39 (36–41)	27 (0–56)	28 (1–43)	35 (29–41)	30 (1–45)	29 (0–55)	29 (0–56)
Vaccination status of measles cases in outbreak 2017/18	Vaccinated with one dose of MCV	1 (25.0%)	4 (80.0%)	43 (52.4%)	6 (54.5%)	1 (50.0%)	8 (57.1%)	34 (57.6%)	97 (54.8)
	Vaccinated with two doses of MCV	-	-	7 (8.5%)	1 (9.1%)	-	4 (28.6%)	1 (1.7%)	13 (7.3)
	Unvaccinated or unknown MCV status	3 (75.0%)	1 (20.0%)	32 (39.0%)	4 (36.4%)	1 (50.0%)	2 (14.3%)	24 (40.7%)	67 (37.9)

MCV- measles-containing vaccine

### Relationships between measles seronegativity and measles outbreak cases by age groups in Vojvodina, Serbia

**Fig 3:**
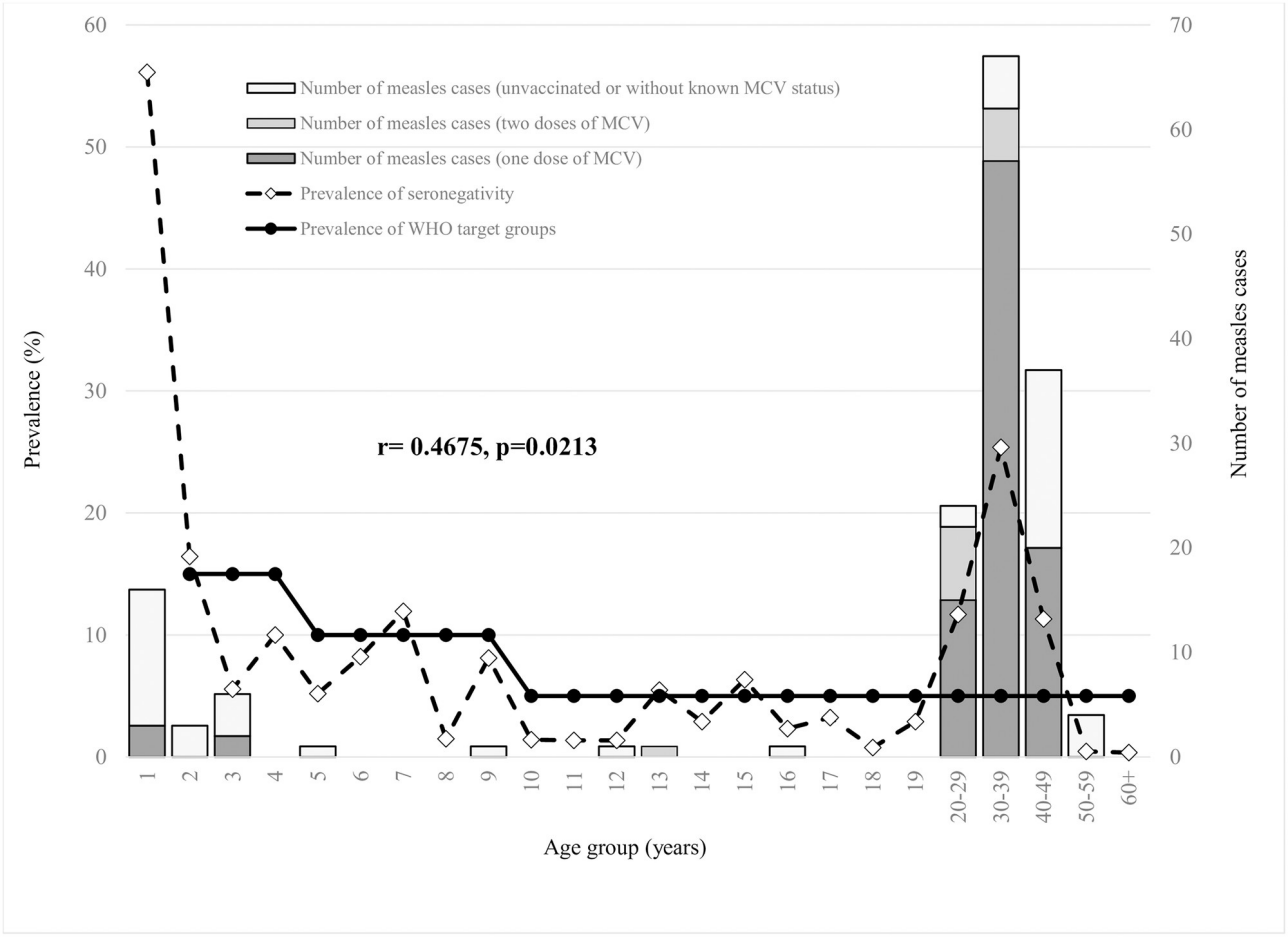
Prevalence of measles seronegativity (2015–2017) and the number of measles outbreak cases (2017–2018) aged 12 months and older by age and vaccination status in Vojvodina, Serbia. MCV: Measles-containing vaccine.

In order to interrupt measles virus transmission, the WHO Regional Office for Europe predicted that the proportion of measles susceptible population should not exceed the following limits: 15% in children aged 2 to 4 years old, 10% in 5 to 9 years old, and 5% in those aged 10 years and older [15, 16]. In participants older than two years, prevalence of measles seronegativity above WHO target levels susceptibility was observed in the following age groups: 2, 7, 13, 15, and among all adults aged between 20 and 49 years. Furthermore, high measles seronegativity (25.4%) was observed in participants aged 30–39 years, in whom also the highest percent (37.9%) of total measles outbreak cases was recorded. Out of the total measles outbreak cases aged 20–29 and 30–39 years, there were 62.5% (15/24) and 85.1% (57/67) previously vaccinated with one dose of MCV, respectively. There was a significant positive correlation between measles seronegativity and the number of reported measles cases aged 12 months and older (r = 0.4675, p = 0.0213) (Fig 3).

Observed by all Districts of Vojvodina, measles seronegativity above WHO target levels [13, 14] was detected in the participants 20–39 years of age, while the prevalence of seronegativity under WHO targets was registered in the age groups of 2–4 and 10–19 years. In addition, measles seronegativity above WHO targets was detected in children aged 5–9 years only in the West Bačka District, and slightly above WHO target levels susceptibility in adults aged ≥40 years only in the South Bačka District (S2 Fig).

## Discussion

To the best of our knowledge, this is the first comprehensive study that allows assessment of the measles seroprevalence data in the context of disease incidence and recent measles outbreaks in Vojvodina, Serbia. In order to predict the risk groups for future outbreak, this paper provides an overview of measles sero-epidemiology in Vojvodina before the last (and ongoing) outbreak of measles.

After the wide introduction of MCV in a certain territory, the changes in epidemiology of measles include a shift of incidence from preschool-aged children to older age groups, and a disease focus in population who did not routinely accept two doses of MCV or had no natural immunity [24]. Although the vaccination against measles is free of charge and obligatory [25, 26], in the last decade, there is a trend of decline in immunization rate mainly for the first dose of MCV in Vojvodina [11], which lead to measles outbreaks with different age-specific distribution among patients [11, 12]. According to experiences from last three small measles outbreak in Vojvodina, the age distribution of cases shifted from preschool-aged children (in 2007) to adolescents (in 2014/15) [11, 12], and adults (ongoing outbreak), respectively. Contrary to the first two mentioned outbreaks (when mostly unvaccinated subjects were infected) [11, 12], in the current outbreak, the majority of reported cases were vaccinated with one dose of MCV according to their age. Similar upward shift in the age of measles cases to older age groups was well described in other settings [27, 28].

One of the possible reasons for the measles outbreak occurrence in our territory is a declining trend of immunization coverage. Various explanations have been given for decreasing trend of immunization in Vojvodina: (1) a drop in timely availability of MMR vaccine in Serbia during 2012–2016, (2) scepticism towards the measles vaccine, which has been supported by negative messages in media and (3) an increasing activity of the anti-vaccination movement in the Balkans countries [11, 29, 30].

Although we did not provide the individual immunization coverage data, and therefore we could not precisely predict the duration of vaccine-induced immunity against measles among participants included in this sero-epidemiological study, the annual measles coverage rate varied widely across Districts. Between 1999 and 2017, the South Bačka District had the lowest coverage with both MCV, where the highest number of measles outbreak cases in 2017/18 (82 of 177, 46.3%) was registered. The possible reason for this obvious decline trend of measles immunization coverage may lie in the fact that during and after the civilian wars in Yugoslavia (1991–1995 and 1999) many of refugees (mostly Serbian and Roma population) settled in the city of Novi Sad which is the main administrative centre of the South Bačka District. Consequently, in the 1990s and 2000s, the city experienced significant population growth. Between 1991 and 2011, in the territories of Novi Sad and the South Bačka District an increasing trend of population was detected, of 28% and 11%, respectively [31]. Large population size in conjunction with the high population density may have a negative impact on the progress of measles elimination [32], which is potentially in line with the aforementioned facts. Furthermore, many of refugees who settled in the South Bačka District were unvaccinated/partly vaccinated or with unknown immunization data against measles. Due to the high number of low-income population who settled in this area after Wars, it was not possible to promptly incorporate them in the available healthcare system; therefore they remained either unvaccinated or incompletely vaccinated and often recognized just only during outbreak occurrence [12, 21]. In addition, the activity of anti-vaccination movement seems to be more present in the South Bačka District than in other Districts of Vojvodina [11].

Successful measles elimination in a certain territory will be accredited when the following criteria are met: vaccine coverage of more than 95% population, continued disease surveillance with incidence rates below 1 per million inhabitants, and the rate of 80% laboratory-confirmed cases among suspected [15, 16]. The other criteria of evidence for verification of elimination of measles are listed in S2 Table [9]. In addition, WHO predicted age specific measles seronegativity target levels for elimination of measles. In this population-based study’s sample of the inhabitants aged ≥12 months, the overall prevalence of measles seronegativity was 11%. In addition, we revealed evidence that the high proportion of measles seronegativity above the WHO elimination target (<5%) in Vojvodina was detected among all participants aged between 20 and 39 years, which strongly confirmed the high burden of disease in this subpopulation in this measles outbreak. Additionally, out of 91 measles outbreak cases aged 20–39 years, 79.1% of them were previously vaccinated with one dose of MCV. Results from our study also showed a significant positive correlation between prevalence of measles seronegativity and all number of measles outbreak cases aged ≥12 months.

According to the prevalence of measles seronegativity in the following age groups: 2–4, 5–9, 10–19, 20–39, and ≥40 years of age, certain country or territory can be classificated into low, medium and high risk for measles outbreaks [33](S3 Table). Given that only one adult age group had the measles susceptibility profile above WHO elimination target, we can assume that Vojvodina can grouped with the regions presenting low susceptibility for outbreaks, similar to Slovakia, Slovenia, Hungary and Sweden [33]. On the other hand, the results of serological surveys from other territories based on the serum banks collected between 1996 and 2004, have identified several countries (Belgium, Bulgaria, England and Wales, Ireland, Romania, Cyprus and Latvia) as those at high risk of measles outbreak [33], which was later confirmed as good prediction for reported large outbreaks in aforementioned countries [34–39].

Apart the age group 20–39 years, the results of our sero-epidemiological study indicated that the prevalence of measles seropositivity gradually increased from the age 12 months to older. However, the proportion of seronegative children was the highest in the youngest (12–23 months) age group (after 12 months of age they should be vaccinated with one dose of MMR vaccine), which can be explained by the delay in the first vaccination against measles. Taking into account the time of MCV introduction in Vojvodina, the low measles seronegativity (3.7%) among participants aged ≥40 years possibly suggests that they acquired immunity against measles through natural infection. For participants aged between 2 and 39 years, we cannot distinguish whether measles seropositivity originated from natural infection or vaccination.

However, it is a known fact that decline in antibody levels shows exponential trend, especially in subjects previously incompletely vaccinated against measles [40]. If we take into account the point of the seroprevalence survey for all subjects aged ≥12 months (during 2015–2017), and the obvious high coverage of the MCV during 1978–1994, the highest prevalence of measles seronegativity in these persons (born between 1977 and 1993), possibly reflects the vanning of immunity after administration of a single dose of the MCV only. Our observation might be consistent with reports of other authors who argue that patients became measles IgG seronegative 14 years following primary vaccination and titres convert to negative between the 3rd and 6th year after immunization [41]. Furthermore, our results also showed that during 2015–2017 seroprevalence study the lowest proportion of seronegativity to measles and the smallest number of measles outbreak cases were detected in participants aged 10–19 (high coverage with both MMR vaccines for subjects aged between 1996–1998 to 2005–2007 cohorts), which probably indicated the solid efficacy and duration of immunity after vaccination with MMR in this age group.

As we mentioned above, the majority of the measles outbreak cases were older than 20 years (132/177; 74.6%). A possible explanation for negligible number of infants and preschool children affected during current measles outbreak can lie in the fact that immediately after the detection of the outbreak, comprehensive control measures were conducted (S3 Fig). Thanks to the massive catch-up campaign for immunization against measles, the coverages for both MMR1 and MMR2 vaccines in Vojvodina increased from 78% and 93% (at the end of 2017) to 90% and 94% (during the first two months of 2018), respectively [42]. Due to the lowest coverage levels of both MMR1 and MMR2 vaccines in the South Bačka District, the immunization campaign was particularly prominent in this area of Vojvodina [43]. An obvious change in parent’s views was observed regarding MMR vaccination which occurred as a result to the direct experience of measles outbreak [44]. The Centre for Disease Control and Prevention of IPHV in collaboration with all medical settings in the South Bačka District and six district coordinators in the local departments of Public Health of Vojvodina monitored close contacts [42].

In addition, guidelines regarding strict respiratory isolation of patients with suspected measles, and vaccination of susceptible hospital staff were forwarded to all hospitals in Vojvodina [44]. As previously described, the health-care workers (HCW) are highly exposed and thus have been affected by many measles outbreaks in the past [45–49]. Due to this fact, we promptly started vaccination of all HCW who worked in the healthcare facilities with high risk of complications from measles (S3 Fig). HCW born before 1971 were considered immune to measles, due to greater circulation of the virus resulting from nearly universal exposure and the development of natural immunity [11, 45]. Before hospitalization of all children younger than 12 months in the hospital setting of high risk for complications of measles, vaccination of unvaccinated mothers or those without the proof of measles IgG seropositivity was recommended also. Furthermore, vaccination with one dose of MMR vaccine was required for all unvaccinated or partly vaccinated medical students involved in hospital practice [50].

Our study had some limitations. First, we did not collect the personal data on history of childhood measles as well as measles vaccination history (there was annual immunization coverage of MCV across Districts of Vojvodina), and therefore we could not differentiate whether measles-specific IgG antibodies was originated from sufficient vaccination or natural infection. Further research could provide the information on duration of vaccine induced immunity after administration of one or two doses of MCV. Second, the samples that were tested equivocal have not been retested, and this could have influenced the observed sex specific difference of measles seropositivity. Standardisation by testing a reference panel was not done. If we supposed that equivocal results were classified as low positive [51, 52], then measles seropositivity might be potentially higher than registered, and differences between sexes would be negligible. Without standardisation, the potential classification of equivocal samples into low positives could be based on the likelihood that an equivocal result indicated declining of the antibody levels rather than vanning of immunity [51, 53]. Taking this finding into account, the overall measles seropositivity in our study reached 89.1%. Third, a portion of children (especially in the South Bačka District) was probably vaccinated by healthcare providers that work in private sector in the last five years, in the same period in which we observed a significant decline in immunization coverage. This population is not recognized by the records available in the Government Health Care Centres that monitors vaccination coverage in our territory. Thus, presented low coverage of MCV in the South Bačka District must be interpreted with caution. Despite the above mentioned potential limitations, we presume that they did not discriminate the main results of our study. Thus, our results are applicable to and representative for the implementation of strategies to sustain measles elimination throughout the Vojvodina.

In conclusion, this study showed age related and geographical differences in the measles seroprevalence in Vojvodina. Despite the small number of outbreak cases, recognition of susceptible age groups is important for targeted supplementary immunization activities. Otherwise accumulation of susceptible individuals within the community, will inevitably lead to the occurrence of the more extensive outbreak in the near future [54]. Educational campaigns should be undertaken in order to improve acceptance and timely MMR vaccination among doctors as well as the general population [55]. Our findings showed that the main susceptible age group for measles transmission in our territory are the subjects aged 20–39 years. In accordance with this, sero-epidemiological study of rubella among women of childbearing age should be addressed in the future. In accordance with the measles seroprofile in Vojvodina, the catch-up campaign for susceptible adults aged 20–39 years, particularly in women before pregnancy seems as an appropriate strategy for progressing toward measles elimination goals [56, 57]. Furthermore, among the youngest age group (12–23 months), the observed seronegativity rate above 55% is concerning. In order to prevent new outbreaks and achieve the elimination of measles in Vojvodina, the vaccination coverage of both MMR vaccines needs to be improved and sustained uniformly in all Districts [58]. Although we did not perform the seroprofiles for infants younger than 12 months, in line with the high level of measles seronegativity in the 20–39 age group, as well as the fact that even infants born to vaccinated or naturally immune women are protected until approximately 4–8 months of age [26, 27, 59, 60], starting first measles vaccination at 9 instead of 12–15 months of age [60–62] should be considered. In order to provide guidance on measles vaccination strategies, data from our study could be used for other regions with similar vaccination history and measles occurrence, where seroprevalence survey data are not available.

## Supporting information

S1 Fig Seroprevalence and geometric mean concentration of measles IgG antibodies by age and sex in Vojvodina, Serbia, 2015–2017. The asterisk (*) indicates p-value < 0.05 for the GMC difference between males and females in certain age group.
(TIF)

S2 Fig Prevalence of measles seronegativity (2015–2017) and the number of measles outbreak cases (2017–2018) by age and Districts of Vojvodina, Serbia. (TIF)

S3 Fig Control measures. MCV: Measles-containing vaccine; HCW: Health-care workers; MMR vaccine: Vaccine against measles, mumps, and rubella.
(TIF)

S1 Table Coverage of MMR1 and MMR2 vaccines by Districts of Vojvodina, Serbia, 1999–2017. (DOCX)

S2 Table Hierarchy of evidence for verification of elimination of measles in a certain territory. (DOCX)

S3 Table WHO target levels susceptibility regarding measles seronegativity by age groups. (DOCX)
