# Supplementary figures and images for: Sero-epidemiological study in prediction of the risk groups for measles outbreaks in Vojvodina, Serbia
Source: PLoS One. 2019 May 9;14(5):e0216219. doi: 10.1371/journal.pone.0216219 (PMC6508608; doi:10.1371/journal.pone.0216219)

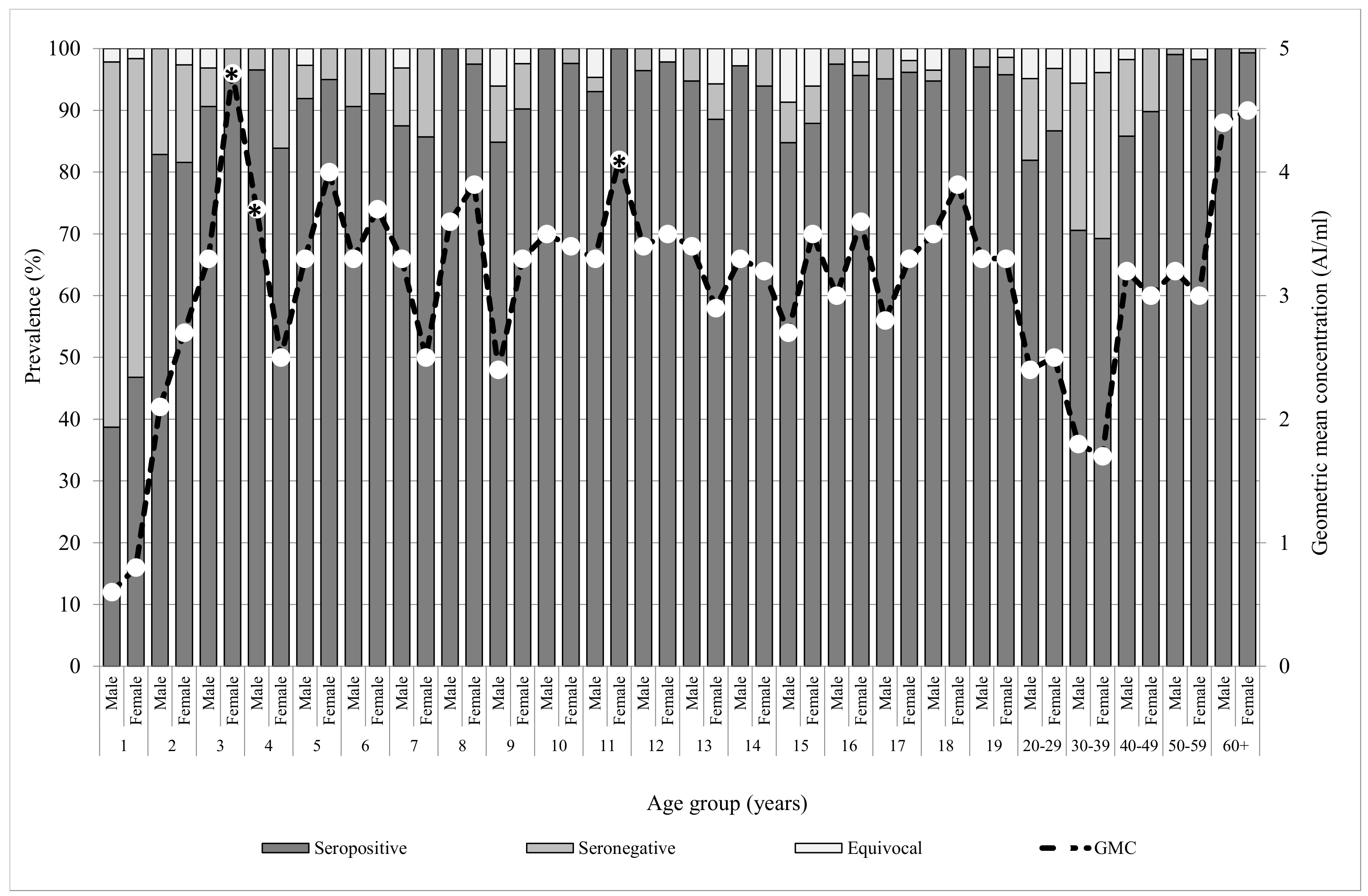

Supplement: S1 Fig — The asterisk (*) indicates p-value < 0.05 for the GMC difference between males and females in certain age group. (TIF) [file pone.0216219.s001.tif]

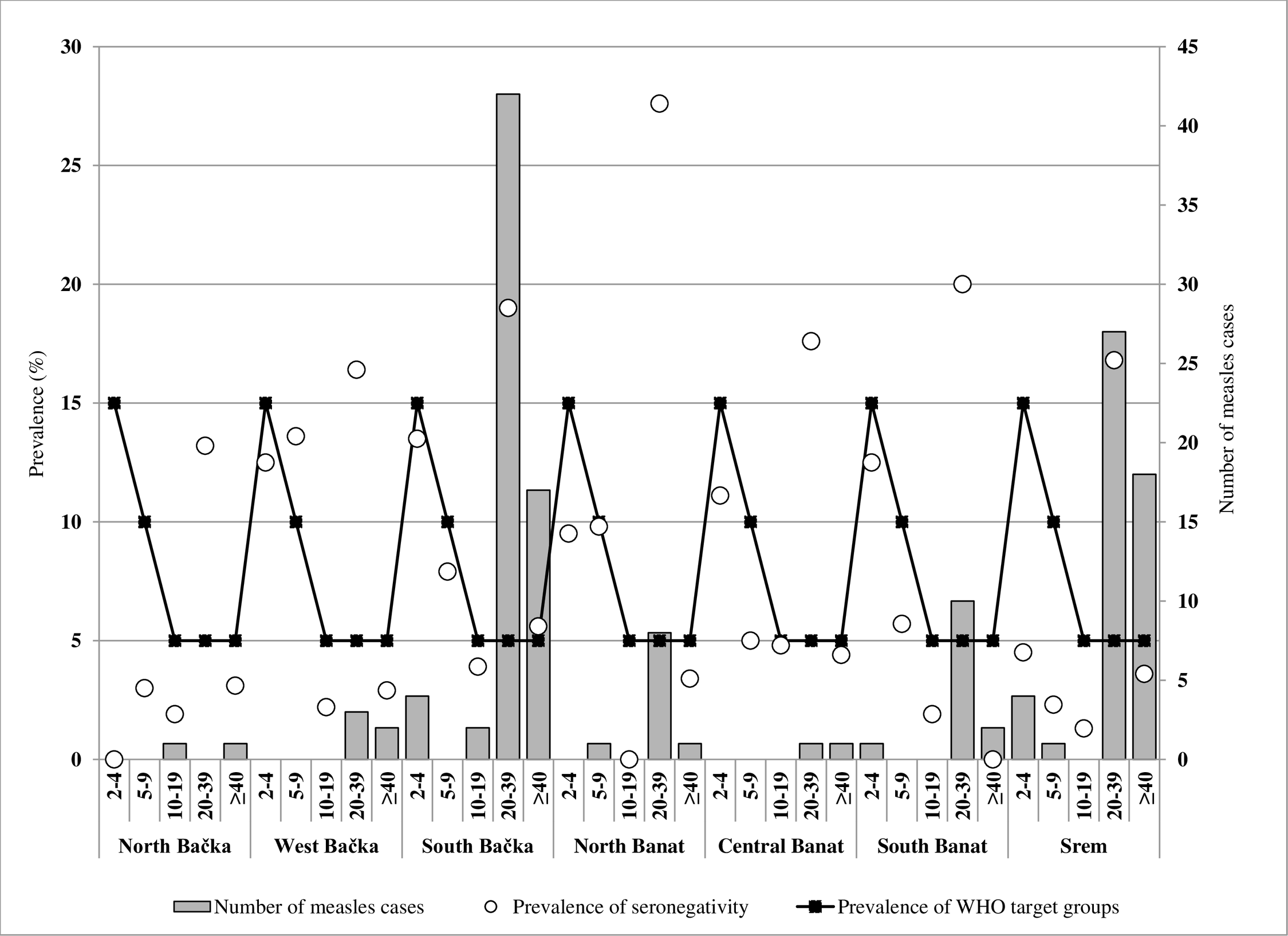

Supplement: S2 Fig — (TIF) [file pone.0216219.s002.tif]

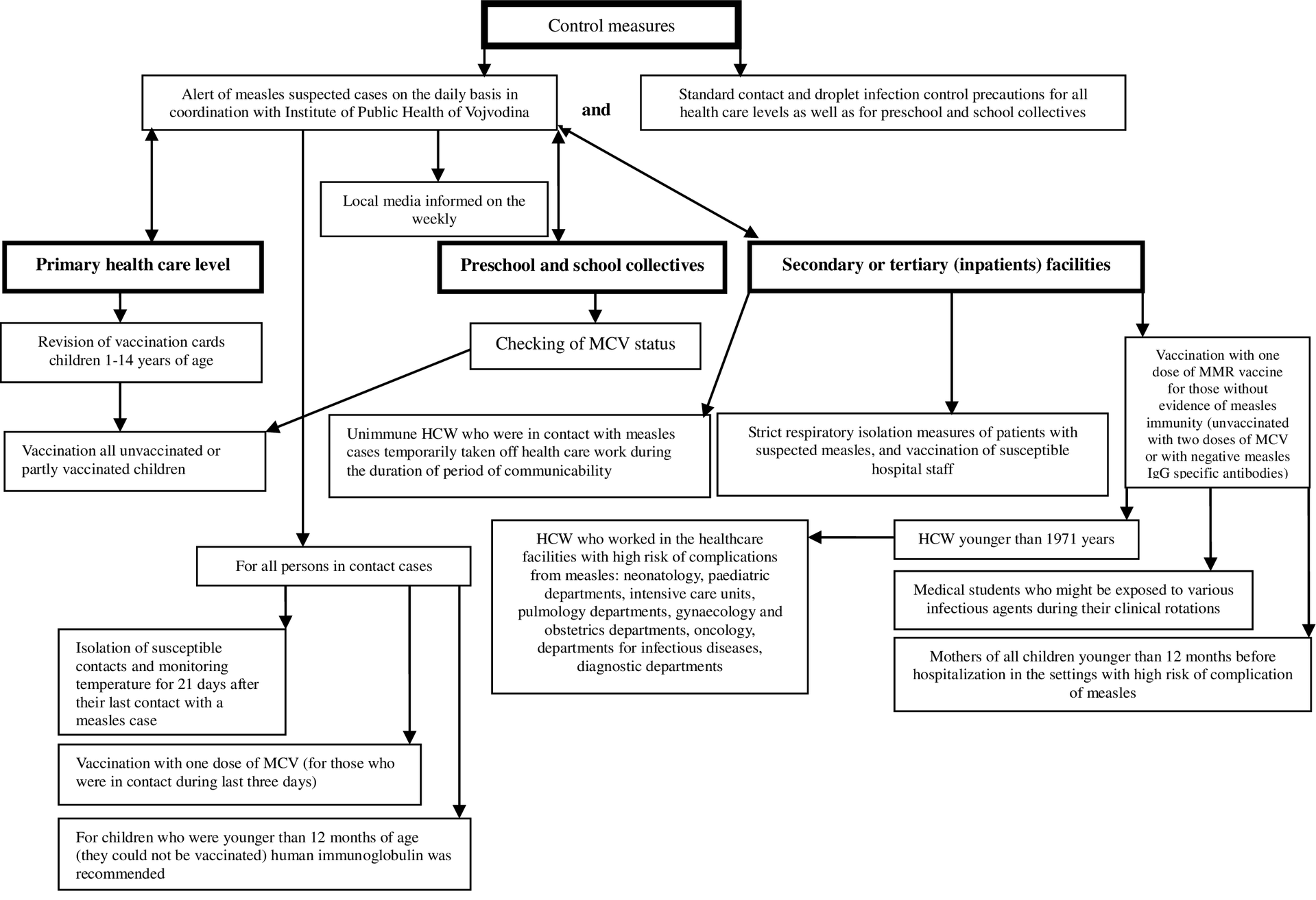

Supplement: S3 Fig — MCV: Measles-containing vaccine; HCW: Health-care workers; MMR vaccine: Vaccine against measles, mumps, and rubella. (TIF) [file pone.0216219.s003.tif]
