# Supplementary material for: Sero-epidemiological study in prediction of the risk groups for measles outbreaks in Vojvodina, Serbia
Source: PLoS One. 2019 May 9;14(5):e0216219. doi: 10.1371/journal.pone.0216219 (PMC6508608; doi:10.1371/journal.pone.0216219)
Supplement: S1 Table — (DOCX) [file pone.0216219.s004.docx]

**S1 Table. Coverage (%) of MMR1 and MMR2 vaccines by Districts of Vojvodina, Serbia, 1999-2017.**

| **District** | | | North Bačka | | West Bačka | | South Bačka | | North Banat | | Central Banat | | South Banat | | Srem | |  |  |
| --- | --- | --- | --- | --- | --- | --- | --- | --- | --- | --- | --- | --- | --- | --- | --- | --- | --- | --- |
| **Measles vaccine** | | | MMR 1  (%) | MMR 2  (%) | MMR 1  (%) | MMR 2  (%) | MMR 1  (%) | MMR 2  (%) | MMR 1  (%) | MMR 2  (%) | MMR 1  (%) | MMR 2  (%) | MMR 1  (%) | MMR 2  (%) | MMR 1  (%) | MMR 2  (%) |  |  |
| **1999** | | | 92.0 | 98.9 | 96.7 | 76.7 | 96.6 | 98.7 | 91.1 | 90.3 | 95.2 | 69.3 | 92.6 | 66.3 | 93.2 | 54.8 |  |  |
| **2000** | | | 80.2 | 47.7 | 94.8 | 68.8 | 70.5 | 52.2 | 91.5 | 83.2 | 87.1 | 79.5 | 86.6 | 98.5 | 85.0 | 56.9 |  |  |
| **2001** | | | 99.8 | 100.0 | 96.5 | 98.2 | 97.8 | 96.9 | 98.2 | 96.8 | 97.2 | 88.6 | 95.8 | 89.4 | 94.4 | 98.4 |  |  |
| **2002** | | | 90.0 | 50.0 | 99.5 | 55.9 | 94.1 | 53.4 | 88.3 | 71.5 | 96.8 | 88.5 | 91.5 | 55.4 | 96.6 | 47.6 |  |  |
| **2003** | | | 99.3 | 98.3 | 98.5 | 98.2 | 93.9 | 91.0 | 95.1 | 71.1 | 98.4 | 63.6 | 96.8 | 90.4 | 98.7 | 99.3 |  |  |
| **2004** | | | 98.4 | 99.2 | 98.0 | 99.4 | 96.4 | 98.7 | 98.2 | 96.1 | 99.0 | 97.8 | 98.0 | 98.4 | 98.9 | 99.0 |  |  |
| **2005** | | | 99.1 | 100.0 | 99.7 | 98.9 | 96.9 | 98.9 | 97.2 | 98.3 | 98.2 | 98.5 | 98.5 | 98.1 | 98.6 | 98.9 |  |  |
| **2006** | | | 100.0 | 90.7 | 99.1 | 100.0 | 96.0 | 99.7 | 98.3 | 99.7 | 94.6 | 93.9 | 93.8 | 97.9 | 95.4 | 98.1 |  |  |
| **2007** | | | 94.8 | 89.8 | 97.7 | 97.6 | 97.1 | 99.9 | 97.4 | 91.9 | 99.2 | 92.1 | 98.1 | 97.2 | 95.7 | 98.7 |  |  |
| **2008** | | | 98.1 | 98.0 | 96.2 | 98.4 | 98.0 | 99.3 | 98.8 | 98.6 | 98.6 | 97.7 | 98.3 | 99.5 | 98.8 | 98.4 |  |  |
| **2009** | | | 98.2 | 97.5 | 97.7 | 94.9 | 97.4 | 99.1 | 96.3 | 92.2 | 95.8 | 94.5 | 98.6 | 99.6 | 97.8 | 97.9 |  |  |
| **2010** | | | 95.3 | 96.5 | 96.8 | 95.0 | 97.3 | 98.8 | 97.9 | 98.5 | 94.0 | 96.0 | 99.3 | 99.3 | 98.7 | 99.6 |  |  |
| **2011** | | | 98.4 | 97.2 | 99.6 | 98.4 | 98.0 | 99.2 | 98.8 | 97.1 | 96.8 | 96.2 | 98.8 | 99.2 | 98.7 | 98.8 |  |  |
| **2012** | | | 96.2 | 98.7 | 94.0 | 97.1 | 83.5 | 86.4 | 91.9 | 97.7 | 96.3 | 95.3 | 98.9 | 99.9 | 90.8 | 97.3 |  |  |
| **2013** | | | 96.7 | 96.4 | 95.5 | 91.4 | 95.0 | 67.0 | 97.6 | 97.6 | 96.6 | 96.4 | 98.9 | 97.9 | 98.6 | 92.8 |  |  |
| **2014** | | | 93.8 | 94.2 | 95.1 | 98.2 | 73.1 | 77.7 | 94.6 | 96.6 | 94.7 | 98.6 | 97.2 | 98.1 | 96.1 | 99.1 |  |  |
| **2015** | | | 93.4 | 93.3 | 97.4 | 97.8 | 91.1 | 72.2 | 97.7 | 95.2 | 81.5 | 91.8 | 88.7 | 91.3 | 84.6 | 85.4 |  |  |
| **2016** | | | 93.8 | 94.7 | 78.4 | 95.1 | 82.4 | 83.3 | 96.8 | 98.1 | 92.1 | 95.9 | 94.9 | 98.3 | 98.1 | 95.9 |  |  |
| **2017** | | | 95.0 | 95.5 | 89.1 | 97.8 | 61.6 | 87.8 | 95.5 | 98.7 | 92.0 | 95.9 | 89.9 | 98.9 | 91.9 | 94.8 |  |  |
|  |  |  |  |  |  |  |  |  |  |  |  |  |  |  |  |  |  |  |
