# Supplementary material for: Sero-epidemiological study in prediction of the risk groups for measles outbreaks in Vojvodina, Serbia
Source: PLoS One. 2019 May 9;14(5):e0216219. doi: 10.1371/journal.pone.0216219 (PMC6508608; doi:10.1371/journal.pone.0216219)
Supplement: S2 Table — (DOCX) [file pone.0216219.s005.docx]

**S2. Hierarchy of evidence for verification of elimination of measles in a certain territory ^a^.**

| Definition | Absence of endemic transmission in a defined geographical area (e.g., region or country) for a period >12 months in the presence of a well-performing  surveillance system |
| --- | --- |
| Criteria | -Verification of interruption of transmission for at least 3 years in the  presence of high quality surveillance  -Maintenance of high quality surveillance systems  -Verification of absence of endemic transmission through viral  surveillance |
| Component  or lines of  evidence | -Epidemiology of measles  -Immunity levels of multiple population cohorts  -Quality of surveillance systems  -Sustainability of the national immunization program  -Molecular epidemiology |
| Surveillance  Quality  Indicators | Examples:  -Rate of reporting discarded non-measles cases at the  national level (Target: ≥2 cases per 100 000 population per year)  -Proportion of suspected cases with adequate specimens for  detecting acute measles infection collected and tested in a  proficient laboratory (Target: >80%) |

**^a^** Adapted from the SAGE Working Group on Measles and Rubella (Draft of 18 October 2012) [9].
