# Supplementary material for: Sero-epidemiological study in prediction of the risk groups for measles outbreaks in Vojvodina, Serbia
Source: PLoS One. 2019 May 9;14(5):e0216219. doi: 10.1371/journal.pone.0216219 (PMC6508608; doi:10.1371/journal.pone.0216219)
Supplement: S3 Table — (DOCX) [file pone.0216219.s006.docx]

**S3 Table. WHO target levels susceptibility regarding measles seronegativity by age groups^a^.**

|  | Measles seronegative (%) | | | | |
| --- | --- | --- | --- | --- | --- |
|  | 2–4  years | 5–9  years | 10–19  years | 20–39  years | ≥40  years |
| WHO target for country or area | < 15 | < 10 | < 5 | < 5 | < 5 |
| Low susceptibility | Met the WHO targets for elimination in all age groups or only missed the target in the one adult age group | | | | |
| Intermediate susceptibility | Met the recommended WHO susceptibility targets in the under 10 year-old age groups (2-4 and 5-9 years of age) but not for older children/adults | | | | |
| High susceptibility | Not met the WHO targets for susceptibility in the under 10 year-old age groups (2-4 and 5-9 years of age) | | | | |

**^a^** Adapted from the WHO recommendation [33].
